# The global vaccine action plan monitoring and evaluation/accountability framework: Perspective

**DOI:** 10.1016/j.vaccine.2020.04.036

**Published:** 2020-07-14

**Authors:** Thomas Cherian, Narendra Arora, Noni E. MacDonald

**Affiliations:** a MMGH Consulting GmbH, Kuerbergstrasse 1, 8049 Zurich, Switzerland; b Executive Director, The INCLEN Trust International, New Delhi, India; c Dalhousie University, IWK Health Centre, Halifax, Nova Scotia, Canada

**Keywords:** Global vaccine action plan, Immunization, Monitoring and evaluation

The Monitoring & Evaluation/Accountability (M&E/A) framework to assess progress was a critical element of the Global Vaccine Action Plan 2012–2020 (GVAP) and was seen by many as a game changer [1]. This article provides perspectives on the design and evolution of the M&E/A framework over the decade, the challenges in assessing progress and holding stakeholders accountable.

## 1 The design and evolution of the M&E/A framework

The GVAP called for leveraging the recommendations of the Commission for Information and Accountability (CoIA) for Women’s and Children’s Health and aligning work, wherever possible, with other accountability efforts. As recommended by the CoIA, a cyclical process of monitoring, review and remedial action was adopted for monitoring the GVAP [2]. At the global level, the World Health Organization (WHO) Strategic Advisory Group of Experts (SAGE) on immunization was responsible for conducting the independent assessment of progress. SAGE was assisted by the Decade of Vaccines Working Group (SAGE DoV WG), which conducted a detailed assessment of progress and prepared a draft assessment report for review by SAGE [3]. While the CoIA recommendations called for monitoring and assessment at the national and global levels, the GVAP M&E/A framework added another level of monitoring at the regional level to accommodate annual reporting to the WHO Regional Committees.

The M&E/A framework aimed to monitor the following three domains:

1. Results (defined as progress against the GVAP goals’ and strategic objectives).
2. Stakeholder commitments to GVAP.
3. Resources invested in vaccines and immunization.

Monitoring results consisted of reviewing progress against the GVAP goals and Strategic Objective (SO) indicators. These were compiled into an annual report by a secretariat hosted by WHO [4]. The indicators were not static but were reviewed and revised periodically by the SAGE DoV WG. The SAGE DoV WG also established new indicators to monitor country capacity for monitoring adverse events following immunization, vaccine stock outs, and integration of immunization with other services.

In addition to the quantitative analysis of data, the secretariat reports also included an assessment of the quality of the data for each indicator as well as a narrative report that included an interpretation of the results and aimed to provide more qualitative information of progress [5].

As called for in the GVAP, monitoring the commitments to GVAP and DoV and tracking resources invested in vaccines and immunization by stakeholders other than the national governments was done jointly with the monitoring process established for the Global Strategy for Women’s and Children’s Health (GSWCH) [6].

## 2 Challenges with assessing progress

Assessment of progress posed several challenges for the SAGE DoV WG and for SAGE.

### 2.1 Data quality

The critical importance of data quality to make meaningful recommendations was recognized and highlighted by the SAGE DoV WG in its very first report [7]. While the quality of data did gradually improve, it remained suboptimal for several indicators (Table 1). For example, the quality of data to assess indicators on two core principles of GVAP, equity and country ownership remain suboptimal. Measurement of inequality focused on vaccination coverage by district (or equivalent administrative level) and by wealth quintile. Not all countries reported subnational coverage. Among those that did, it was difficult to assess the validity of the reported coverage rates. A very simple rule was developed to assess validity: if the WHO-UNICEF estimates of national immunization coverage (WUENIC) and administrative coverage reported by countries are the same or if the WUENIC was ≥90%, the reported district coverage was considered valid. However, this rule had its limitations [8]. Data on coverage by wealth quintile were only available from 84 (43%) countries and only 28 had two data points during the decade to assess progress [5]. The uncertainty ranges around the coverage estimates, stratified by wealth quintile, from household surveys were wide, making statistical comparisons difficult.

**Table 1:** Indicators that posed challenges for monitoring and interpretation.

Goal/ Strategic Objective	Indicator	Challenge
G3 Meet vaccination coverage targets in every region, country and community	G3.1 Reach 90% national coverage and 80% in every district or equivalent administrative unit with DTP3*	Not all countries reported district coverage and measures to validate coverage estimates were inadequate
	G3.2. Reach 90% national coverage and 80% in every district or equivalent administrative unit for all vaccines in national programmes, unless otherwise recommended	Countries did not report district coverage with vaccine doses other than DTP3
G5 Exceed the millennium development goal 4 target for reducing child mortality	G5.1 Under 5 morality rate per 1000 live births	Contribution of vaccination to mortality reduction was difficult to estimate
SO1 All countries commit to immunization as a priority	SO1.1. Domestic expenditures for immunization per person targeted	Reported domestic expenditures for immunization varied widely from year to year without explanation and were not congruent with data reported in the health accounts data base
	SO1.2. Presence of an independent technical advisory group that meets defined criteria	Did not measure functionality, outputs and added value of the advisory groups
SO2 Individuals and communities understand the value of vaccines and demand immunization both as a right and a responsibility	SO2.1. Percentage of countries that have assessed (or measured) the level of confidence in vaccination at subnational level	The indicators only assessed confidence in vaccination, which is only one component of demand, with variable understanding of what confidence meant. Hence, it was unclear what was being measured
	SO2.2. Percentage of un- and under-vaccinated in whom lack of confidence was a factor that influenced their decision	
SO3 The benefits of immunization are equitably extended to all people	SO3.1. Percentage of districts with 80% or greater coverage with DTP3	Same as G3.1
	SO3.2. Reduction in coverage gaps between wealth quintiles and other appropriate equity indicator(s)	Data available only from limited set of countries with even fewer countries with two data points in the decade
SO4 Strong immunization systems are an integral part of a well-functioning health system	SO4.3. Immunization coverage data assessed as high quality by WHO and UNICEF	This indicator was dropped since no suitable measure could be found
	SO4.4. Number of countries with case-based surveillance for vaccine-preventable diseases	Measured only the presence but not the quality of surveillance
	SO4.5. Number of countries reporting at least 10 AEFI** cases per 100 000 surviving infants.	Did not permit assessment of reporting of severe AEFI
SO6 Country, regional and global research and development innovations maximize the benefits of immunization.	SO6.3. Progress towards institutional and technical capacity to carry out vaccine clinical trials	Was limited to the number of trials registered in clinical trial registries with no assessment of trial outputs
	SO6.5. Number of vaccine delivery technologies (devices and equipment) that have received WHO prequalification against the 2010 baseline	Results did not lead to any meaningful recommendations.

*DTP3 = coverage with three doses of diphtheria-tetanus-pertussis containing vaccines; **AEFI = Adverse Events following vaccination.

The key indicator used to measure country ownership was national domestic expenditures on immunization. Wide, unexplained year-to-year fluctuations in expenditures were noted in several countries, making it difficult to monitor and interpret trends.

### 2.2 Framing appropriate indicators

Certain indicators proved to be difficult to frame. Of the original 27 indicators, 14 posed challenges in assessment and/or interpretation (Table 1). While the assessment of demand for and acceptance of vaccination was important, given increasing vaccine hesitancy being reported from all regions, development of appropriate indicators proved to be challenging. Initial country reports showed a lack of understanding of the indicator [9], leading to a change in the definition that focused on hesitancy and permitted analysis of the data [10]. Similarly, while the indicator for integration suggested failures with integrating immunization with other public health programmes the reasons for failure were not evident. Both indicators highlighted the limitations of a single global indicator to capture heterogenous complex contextual issues and lead to meaningful recommendations. While the indicators drew attention to problem areas, they were not in themselves enough to understand the root causes for success or failure, which limited the capacity of the SAGE DoV WG and SAGE to frame actionable recommendations for corrective actions. During the latter part of the decade, the secretariat progress reports included country case studies that consolidated the findings from different programme reviews in each of several priority countries, which led to a better understanding of the determinants of success and failures, but was limited to a select number of countries each year.

### 2.3 Monitoring the contributions of non-Governmental stakeholders

Assessment of “results” was meant to be complemented by monitoring of stakeholder commitments and resources invested in immunization in conjunction with the monitoring process for the GSWCH. However, it was difficult to disaggregate the information on immunization from broader commitments to maternal and child health in the country compacts. Similarly, information on expenditures on immunization were only available from 36 countries through the System of Health Accounts in 2018 and the quality of data remained uncertain [4]. Tracking of resources for immunization through the GSWCH only provided data from Gavi and the Global Polio Eradication Initiative for a single report in 2015, though subsequent studies were able to collect data from a broader set of agencies for the period 2000 to 2016 [11].

## 3 The accountability processes

Accountability was one of the key components of the M&E/A framework and process. In adopting the cyclical process of monitoring, independent review and corrective actions as recommended by the CoIA, the assumption was that the various stakeholders would be accountable for acting on the recommendations [2]. To achieve this end it was important to maintain the independence of the review and assessment process and establish mechanisms for holding stakeholders accountable.

### 3.1 Maintaining the independence of the review process

The task of collecting, analysing and presenting the information on progress was the role of the GVAP secretariat agencies^1^ that were also important stakeholders of the GVAP. In order to maintain the integrity of the independent review process, the GVAP secretariat limited its role to the collection and analysis of the data for each indicator along with a narrative report. The assessment process was carried out by the SAGE DoV WG, which was provided with a medical writer to assist with the preparation of the report that went to SAGE and thereafter, with revisions, submitted as an addendum to the WHO secretariat report to the WHO Executive Board and World Health Assembly (WHA) annually.

### 3.2 Holding stakeholders accountable

While the GVAP broadly described the roles and responsibilities of different stakeholders, none of the stakeholders made specific commitments for which they could be held accountable. Beyond the expectations that stakeholders would implement SAGE recommendations there were no measures for accountability.

Meetings were held with country delegations at the WHA and regional meetings to highlight progress and challenges and draw attention to the recommendations for corrective actions. Country report cards were also developed and distributed to country delegations [12].

The actions taken by the GVAP secretariat agencies in response to SAGE recommendations were reported annually to the SAGE DoV WG and periodically included in the secretariat reports to the WHA. Time and resources did not permit monitoring of actions taken in response to SAGE recommendations by countries or other non-governmental stakeholders.

## 4 Lessons for monitoring, evaluation and accountability at the global level for the next decade

Our experience provides a few important lessons that have informed the development of the monitoring and accountability framework for the Immunization Agenda 2030 (IA2030).

Data quality may remain an impediment to monitoring and accountability in the next decade, unless SAGE recommendations on quality and use of data are acted upon [13].

Global indicators highlight problems, but their root causes are contextual; further evaluation is required at the country level to make actionable recommendations.

Holding global or regional level stakeholders accountable may be achievable through the global and regional M&E/A process, but there should be very specific and measurable deliverables that the stakeholders agree to be held accountable for, accompanied by indicators to assess if they are met.

Country level accountability is best administered at the country level, with oversight and support being provided for this at the regional level. Here again, roles and responsibilities of each stakeholder should be clearly defined, accompanied by measurable indicators.

Aspirational goals that were considered unachievable and lack of commensurate resources to implement recommendations made it challenging to hold countries accountable.

Dedicated financial resources will be required to fully implement the M&E/A process at the global, regional and country levels. In addition, many countries, especially those not meeting targets, will require technical and financial support to establish and implement robust M&E/A processes.

## Declaration of Competing Interest

The authors declare the following financial interests/personal relationships which may be considered as potential competing interests: [Thomas Cherian assisted the Decade of Vaccines (DoV) Collaboration Delivery Working Group and served on the M&E/A Secretariat from 2012 to 2017 as an employee of WHO. Narendra Arora (2012–2017) and Noni MacDonald (2017–2019) served as chairpersons of the SAGE DoV WG and currently serve on the Task Force for developing the M&E framework for the Immunization Agenda 2030 (IA2030). All authors attest they meet the ICMJE criteria for authorship.].

## Funding

This work was supported by a grant from the Bill & Melinda Gates Foundation to the World Health Organization [grant number OPP1128274].

## Footnotes

1 Bill & Melinda Gates Foundation, Gavi, the Vaccine Alliance, the US National Institute for Allergy and Infectious Diseases, United Nations Children’s Fund (UNICEF) and WHO.
